# Attempted Suicide in a Parkinsonian Patient Treated with DBS of the VIM and High Dose Carbidopa-Levodopa

**DOI:** 10.1155/2019/2903762

**Published:** 2019-03-26

**Authors:** Ayotunde Ayobello, Brian Saway, Michael Greenage

**Affiliations:** Virginia Tech Carilion School of Medicine and Research Institute, USA

## Abstract

**Introduction:**

Parkinson's disease (PD) is a complex disease that is often treated with dopaminergic medications such as carbidopa-levodopa and now with innovative interventions such as deep brain stimulation (DBS). While PD frequently presents with depression and apathy, research must elucidate whether its treatment modalities have an additive or synergistic effect that can lead to an increased suicide risk. DBS has been associated with depression, behavioral changes, and suicidality while dopaminergic treatment has also been shown to cause behavioral changes such as hypersexuality and impulsivity. Considering the now frequent practice of utilizing both DBS and carbidopa-levodopa to treat PD, it is crucial to understand how to properly manage PD patients who are displaying this overlap in symptomology.

**Case Report:**

A 56-year-old Caucasian male with a 6-year diagnosis of PD who was being treated with high dose carbidopa-levodopa and left DBS of the ventral intermediate nucleus (VIM) presented after a suicide attempt. The patient was found to be severely depressed and had exhibited behavioral changes in the weeks leading up to the attempt. Imaging was performed to assess positional changes of DBS and carbidopa-levodopa dosage adjusted while under close observation in the inpatient unit. The patient was started on fluoxetine to treat the depressive symptoms and was eventually discharged with close monitoring.

**Discussion:**

With PD and DBS being associated with behavioral changes and depressive symptoms and carbidopa-levodopa therapy being linked to behavioral changes such as impulsivity, it is important that these patients be closely monitored and research analyzes how these factors may interact and lead to an increased risk of suicide. Furthermore, when symptoms appear, a clear protocol must be established on managing these patients. We therefore recommend an approach that utilizes imaging to assess any changes in DBS placement, dose management of carbidopa-levodopa, and behavior monitoring in an inpatient setting.

## 1. Report

### 1.1. Introduction

Parkinson's disease (PD) is a complex neurodegenerative disorder that can leave patients both physically and mentally disabled [1]. While the dopaminergic and neuronal degeneration seen in Parkinson's disease classically presents with motor symptoms such as resting tremor, rigidity, and postural instability, less discussed are the psychiatric symptoms of depression, impulsivity, and suicidality [1–5]. Fortunately, a number of treatment modalities including dopamine-replacement therapies exist for PD, providing effective but often short-lived relief from symptoms [6]. The refractory nature of symptoms often leads patients to seek less effective second-line pharmaceutical treatments, and once these fail, they resort to the innovative but invasive deep brain stimulation (DBS) therapy. While DBS of the subthalamic nucleus (STN), globus pallidus interna (GPI), and ventral intermediate nucleus (VIM) has been shown to dramatically decrease the motor symptoms associated with PD [7, 8], there is conflicting evidence linking DBS to long term behavioral and emotional symptoms [9–15]. This evidence is further confounded as the disease pathology itself and its pharmacologic interventions have been well documented in their ability to cause similar behavioral changes [2–4, 16]. The management of PD patients displaying psychiatric symptoms can be therefore challenging as the source of manifesting symptoms can be often ambiguous. Here we describe the management and treatment of a PD patient treated with both high dose carbidopa-levodopa and DBS of the ventral intermediate nucleus (VIM) that presented to the inpatient psychiatric unit after a suicide attempt and significant behavioral changes.

## 2. Case Description

The patient is a 56-year-old married, retired Caucasian male with a 6-year history of PD. He previously worked in a scientific lab at a large academic institution and seven years prior to presentation, he began to experience a right-handed tremor that increased in severity over several months. He was evaluated by neurology after which he was diagnosed with PD. He was treated with various dopaminergic medications, including carbidopa-levodopa, with poor symptom relief. Due to poor response to medications, the patient decided to elect for DBS treatment. He received DBS in the left VIM 3 years prior to presentation and experienced significant relief of his right-handed tremor. Over time, the patient began to develop a worsening left-handed tremor and bradykinesia that required restarting treatment with carbidopa-levodopa. The dosage of carbidopa-levodopa was increased over the years for continued control of his Parkinsonian symptoms. The settings of DBS were also increased for continued control of the right-handed tremor. During this time, the patient began to experience changes in behavior including apathy and poor communication skills in social settings. One month prior to presentation, family members noted that the patient began to display uncharacteristic hypersexuality, anger, and impulsivity. The patient was subsequently found by family members in the car garage after what appeared to have been a suicide attempt by carbon monoxide poisoning. According to the patient and family members, this was the patient's first suicide attempt. Emergency services were able to arrive on time to transport the patient to the ED where he was found to have oxygen saturation of 94%, and an arterial blood gas study found a carboxyhemoglobin level of 49.3 (normal <2). Neurology and psychiatry were consulted to evaluate the patient's neurological and mental status. Neurology (internal medicine) found that the patient was taking 3 tablets of carbidopa-levodopa 25-100 (25 mg of carbidopa and 100 mg of levodopa) 5 times a day and was concerned that the behavioral changes may be due to the high dose of carbidopa-levodopa and attempted to reduce the dose to 1 tablet 3 times a day. However, the patient developed acute neck dystonia and the carbidopa-levodopa dose was increased up to 1 tablet 5 times a day. The patient was subsequently admitted to inpatient psychiatry.

Upon intake to inpatient psychiatry, on the lowered dose of carbidopa-levodopa, the patient was noted to display low levels of positive affectivity and was chronically nihilistic. Additionally, he displayed a lack of affect, the lack of appreciation or interest in the nearly completed suicide attempt, and a lack of appreciation of how out of character this was for him. He appeared to have poor insight into his current situation and was often superficial and vague with his responses. No significant behavioral issues were noted.

Investigation into the cause of the patient's behavioral decline and eventual suicide attempt subsequently began with a systematic approach to all the possible offending agents. Both DBS and carbidopa-levodopa are well documented independent causes of behavioral changes. The pathology of PD itself is also associated with behavioral changes such as hypersexuality, pathological gambling, and mood swings. Hypersexuality in particular appears to be a common variable associated with all three factors. We therefore approached each risk factor independently.

### 2.1. Carbidopa-Levodopa, Hypersexuality, and Impulsivity

**Figure 1:**
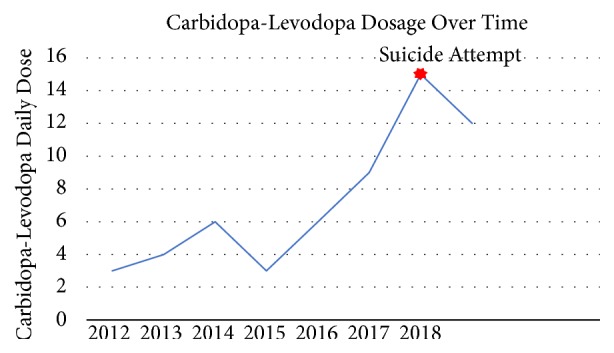
Graph depicting the dosage increase of carbidopa-levodopa over time and the dosage of carbidopa-levodopa at time of suicide attempt.

It is known that a dose dependent association exists between carbidopa-levodopa and altered behavior including hallucinations and/or hypersexuality [16, 17], quite a contrast to the baseline depression and psychological akinesia seen in PD. Increased dopaminergic activity is indeed the hallmark of illicit substances such as cocaine and amphetamines so the development of behavioral changes is therefore not surprising with high doses of carbidopa-levodopa. In this case, Mr. C clearly demonstrated these behavioral changes as evidenced by his increasingly bizarre sexual advances on his wife which were occurring with higher frequency as his dose of medication increased.  Figure 1 shows the gradual increase in carbidopa-levodopa dosing over the years for the patient.

Figure 1 clearly shows the increase in carbidopa-levodopa required to treat the patient's Parkinsonian symptoms and the high daily dose the patient was taking when he made his suicide attempt. The decision was therefore made to decrease his carbidopa-levodopa dosage from 1 tablet 5 times a day to 1 tablet 4 times a day and to continue to monitor his behavior on the inpatient unit. The patient was also started on fluoxetine as depression and apathy are known to occur when the dosage of carbidopa-levodopa is changed or stopped. It was believed that the patient's PD would also benefit from the mild dopaminergic nature of fluoxetine.

The patient did well on the reduced dose of carbidopa-levodopa and did not exhibit any strange behavior, hypersexuality, or impulsivity while on the unit. He also did not endorse any suicidal ideation, thoughts, or plans and appeared to tolerate fluoxetine well.

### 2.2. DBS, Depression, and Suicidality

Subthalamic nucleus (STN) stimulation is considered an established surgical treatment for Parkinson's disease (PD). Episodes of depression and suicide have however been reported in patients treated with DBS of STN, notably when stimulation targets the inferior part of the STN which can induce acute depression symptoms [18]. As the stimulation improves motor symptoms, patients are more able to attempt suicide. Limited data however exists regarding VIM stimulation especially with regard to its cognitive and behavioral effects. Very little data also exists on the impact of positional changes of the device itself over the years. This was especially relevant for the patient after family members reported a mild concussion he had suffered a few months prior.

**Figure 2:**
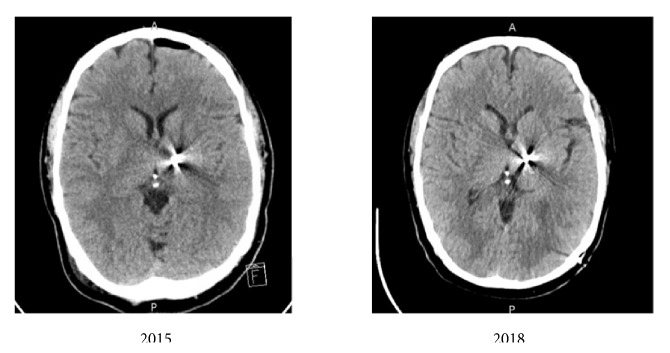
CT of head from 2015 and 2018 demonstrating no obvious movement of DBS device or pathology surrounding device.

The decision to obtain a CT scan was therefore justified with the aim of ruling out potential positional changes within the past 3 years.  Figure 2 shows CT scans from 2015 and 2018.

Upon comparison with a CT from the time of DBS placement, radiology determined that there was no significant change in DBS position and it was concluded that a positional change was not the cause of the patient's behavior. It is therefore reasonable to assume that VIM stimulation by itself might have been, despite the high doses of carbidopa-levodopa, the cause of his depression and eventual suicide attempt. This was especially important to note as the patient and his neurologist were beginning to consider DBS of the right VIM to control his worsening left-handed tremor.

The patient's family played a large role in his treatment as they visited daily to speak to both the patient and his care team. They also agreed that the patient's behavior had improved from the time of presentation. It was decided, after 8 days of inpatient stay, that he was ready for discharge with close follow-up with both neurology and psychiatry.

## 3. Discussion

Behavioral changes, depression, and suicidality have been reported independently in both patients suffering from PD and PD patients surgically treated with DBS. Patients being treated with carbidopa-levodopa have also been known to exhibit behavioral changes [1, 4, 9–14, 16]. However, this case is unique because there is no literature regarding the management and outcome of a patient suffering from behavioral changes and suicidality with all three of these risk factors occurring simultaneously. While one study found that 30% of patients suffering from PD had active suicidal or death ideation [3], another study found a 4.3% rate of suicide for patients treated with DBS for movement disorders [9]. Along with the rising rate of PD patients electing for DBS surgery while simultaneously being treated with carbidopa-levodopa, which has a known side effect of behavioral changes and impulsivity [16], it is hypothesized that these behaviorally activated patients may have a significant increase in risk of suicidality as seen in our patient as these patients aberrantly act on their depressive symptoms. These statistics support the notion that it is imperative that more research be conducted to understand whether these three components have a synergistic or additive effect on each other and how to manage this growing patient population. As new brain regions are being targeted for DBS, this case calls for further research elucidating the potential psychiatric side effects of stimulation of various brain regions such as the VIM. Lastly, and most importantly, this case supports the argument for close psychiatric monitoring of PD patients treated with these two modalities to prevent adverse psychiatric events such as depression and suicide.
